# Predictors of Sexual Dysfunction in Unipolar Depression: A Register-Based Nested Case-Control Study

**DOI:** 10.1192/j.eurpsy.2026.10604

**Published:** 2026-07-31

**Authors:** J. Isung, P. Karlsson, O. Tankaya, V. Johansson, K. Gembert, J. Reutfors

**Affiliations:** 1 Karolinska Institutet, Stockholm, Sweden; 2 J&J Innovative Medicine, Raritan, United States; 3 Umeå University, Umeå, Sweden

## Abstract

**Introduction:**

Sexual dysfunction (SD) is a frequent comorbidity and a potential adverse consequence of treatment in depression. Antidepressants - particularly those with serotonergic effects - are known to contribute to sexual side effects, yet SD often remains underrecognized in clinical settings. Despite high self-reported rates in survey-based studies, large-scale data on clinically recorded SD and associated treatment patterns in depressed populations remain limited. Understanding predictors of SD at the population level is crucial for improving detection, guiding therapeutic decisions, and informing patient care.

**Objectives:**

To identify pharmacological and clinical predictors of SD in men with depression using Swedish nationwide registers.

**Methods:**

In a cohort of 73,566 men aged 18–65 with incident depression (ICD-10: F32/F33) in Sweden, we identified 1,483 SD cases (ICD-10: F52.0–52.3 or ATC: G04BE) within one year from the depression diagnosis, matched 1:5 to 6,704 controls by age, time since depression diagnosis, and region of residence. Predictors were prescriptions of antidepressants (serotonin reuptake inhibitors (SSRIs), serotonin and norepinephrine reuptake inhibitors (SNRIs), tricyclic antidepressants, bupropion, mirtazapine), atypical antipsychotics (quetiapine, olanzapine, aripiprazole), and clinical variables (e.g., depression severity, psychiatric comorbidities, suicide attempts). Conditional logistic regression models were used to estimate adjusted odds ratios (aORs) with 95% confidence intervals (CI).

**Results:**

The use of SSRIs (aOR 1.37, 95% CI 1.17–1.61), SNRIs (aOR 1.81, 95% CI 1.47–2.23), and multiple antidepressants (aOR 1.71, 95% CI 1.44–2.04) was statistically significantly associated with SD. In contrast, the use of mirtazapine (aOR 1.01, 95% CI 0.79–1.29), bupropion (aOR 1.56, 95% CI 0.91–2.69), and TCAs (aOR 1.34, 95% CI 0.85–2.09) showed no statistically significant association. Among antipsychotics, only quetiapine was statistically associated with SD (aOR 1.57, 95% CI 1.07–2.31). No statistically significant associations were found for depression severity, psychiatric comorbidities, or recent suicide attempts.

**Conclusions:**

This national case-control study found that serotonergic antidepressants were associated with higher odds of recorded SD in men with depression, whereas bupropion and mirtazapine showed no significant associations. No clear associations were observed with baseline clinical characteristics. While causality cannot be inferred, findings suggest pharmacological factors may influence SD risk and underscore the importance of addressing sexual health in depression management.

**Disclosure of Interest:**

J. Isung Grant / Research support from: Research support from: Affiliated with/employed at the center for Pharmacoepidemiology, Karolinska Institutet, which receives grants from several entities (pharmaceutical companies, regulatory authorities, contract research organizations) for the performance of drug safety and drug utilization studies., P. Karlsson Employee of: Research support from: Affiliated with/employed at the center for Pharmacoepidemiology, Karolinska Institutet, which receives grants from several entities (pharmaceutical companies, regulatory authorities, contract research organizations) for the performance of drug safety and drug utilization studies., O. Tankaya Employee of: Employee of J&J Innovation. The work on this study was part of the employment., V. Johansson Grant / Research support from: Research support from: Affiliated with/employed at the center for Pharmacoepidemiology, Karolinska Institutet, which receives grants from several entities (pharmaceutical companies, regulatory authorities, contract research organizations) for the performance of drug safety and drug utilization studies., K. Gembert Employee of: Research support from: Affiliated with/employed at the center for Pharmacoepidemiology, Karolinska Institutet, which receives grants from several entities (pharmaceutical companies, regulatory authorities, contract research organizations) for the performance of drug safety and drug utilization studies., J. Reutfors Grant / Research support from: Research support from: Affiliated with/employed at the center for Pharmacoepidemiology, Karolinska Institutet, which receives grants from several entities (pharmaceutical companies, regulatory authorities, contract research organizations) for the performance of drug safety and drug utilization studies.

